# Intra- and Inter-Scanner Reliability of Voxel-Wise Whole-Brain Analytic Metrics for Resting State fMRI

**DOI:** 10.3389/fninf.2018.00054

**Published:** 2018-08-21

**Authors:** Na Zhao, Li-Xia Yuan, Xi-Ze Jia, Xu-Feng Zhou, Xin-Ping Deng, Hong-Jian He, Jianhui Zhong, Jue Wang, Yu-Feng Zang

**Affiliations:** ^1^Center for Cognition and Brain Disorders, Institutes of Psychological Sciences, Hangzhou Normal University, Hangzhou, China; ^2^Zhejiang Key Laboratory for Research in Assessment of Cognitive Impairments, Hangzhou, China; ^3^Center for Brain Imaging Science and Technology, Key Laboratory for Biomedical Engineering of Ministry of Education, College of Biomedical Engineering and Instrumental Science, Zhejiang University, Hangzhou, China

**Keywords:** inter-scanner reliability, intra-scanner reliability, ALFF, PerAF, ReHo, voxel-wise whole-brain analysis

## Abstract

As the multi-center studies with resting-state functional magnetic resonance imaging (RS-fMRI) have been more and more applied to neuropsychiatric studies, both intra- and inter-scanner reliability of RS-fMRI are becoming increasingly important. The amplitude of low frequency fluctuation (ALFF), regional homogeneity (ReHo), and degree centrality (DC) are 3 main RS-fMRI metrics in a way of voxel-wise whole-brain (VWWB) analysis. Although the intra-scanner reliability (i.e., test-retest reliability) of these metrics has been widely investigated, few studies has investigated their inter-scanner reliability. In the current study, 21 healthy young subjects were enrolled and scanned with blood oxygenation level dependent (BOLD) RS-fMRI in 3 visits (V1 – V3), with V1 and V2 scanned on a GE MR750 scanner and V3 on a Siemens Prisma. RS-fMRI data were collected under two conditions, eyes open (EO) and eyes closed (EC), each lasting 8 minutes. We firstly evaluated the intra- and inter-scanner reliability of ALFF, ReHo, and DC. Secondly, we measured systematic difference between two scanning visits of the same scanner as well as between two scanners. Thirdly, to account for the potential difference of intra- and inter-scanner local magnetic field inhomogeneity, we measured the difference of relative BOLD signal intensity to the mean BOLD signal intensity of the whole brain between each pair of visits. Last, we used percent amplitude of fluctuation (PerAF) to correct the difference induced by relative BOLD signal intensity. The inter-scanner reliability was much worse than intra-scanner reliability; Among the VWWB metrics, DC showed the worst (both for intra-scanner and inter-scanner comparisons). PerAF showed similar intra-scanner reliability with ALFF and the best reliability among all the 4 metrics. PerAF reduced the influence of BOLD signal intensity and hence increase the inter-scanner reliability of ALFF. For multi-center studies, inter-scanner reliability should be taken into account.

## Introduction

With its advantages of being non-invasive, fairly good spatial as well as temporal resolution, and very similar design across studies, resting-state functional magnetic resonance imaging (RS-fMRI) of blood oxygenation level dependent (BOLD) technique is promising for clinical research to reveal abnormal spontaneous brain activity. Therefore, intra- and inter-scanner reliability is essential in RS-fMRI studies.

In recent years, the intra-scanner reliability (i.e., test-retest reliability) of many metrics in RS-fMRI has been investigated, such as the amplitude of low frequency fluctuations (ALFF) (Zuo et al., 2010a; Li et al., 2012; Zuo and Xing, 2014; Somandepalli et al., 2015; Zou et al., 2015), regional homogeneity (ReHo) (Li et al., 2012; Zuo et al., 2013; Somandepalli et al., 2015), seed-based functional connectivity (FC) (Shehzad et al., 2009; Patriat et al., 2013; Pannunzi et al., 2017), group-level dual regression independent component analysis (drICA) (Zuo et al., 2010b), voxel-mirrored homotopic connectivity (VMHC) (Zuo et al., 2010c), graph theory (Wang et al., 2011; Braun et al., 2012; Tomasi and Volkow, 2014; Andellini et al., 2015; Aurich et al., 2015). Generally, most of these metrics showed moderate to high intra-scanner reliability.

While many studies have investigated the intra-scanner reliability of RS-fMRI metrics, only one article, to the best of our knowledge, studied the inter-scanner reliability of BOLD RS-fMRI. Jann and colleagues scanned BOLD RS-fMRI data on two same type of scanners (3T Siemens TIM Trio) with identical scanning parameters (Jann et al., 2015). They identified five networks with ICA and computed voxel-wise intra-class correlation (ICC) coefficient within each network. The authors found moderate to high inter-scanner reliability. One limitation for ICA is that only a limited number networks are analyzed. In practice, to map the inter-scanner reliability of every voxel in the whole brain, i.e., “voxel-wise whole-brain” (VWWB) analysis, is needed.

Amplitude of low frequency fluctuation, ReHo, and degree centrality (DC) are three most commonly used methods of VWWB analysis (Zang et al., 2015). The intra-scanner reliability or test-retest reliability of the three metrics have been widely investigated (Zuo et al., 2010a; Li et al., 2012; Liao et al., 2013). However, the inter-scanner reliability of the three metrics has not been thoroughly studied yet. Therefore, the main purpose of the current study was to systematically measure the intra- and inter-scanner reliability of the 3 RS-fMRI metrics. Lower reliability might be due to either random variance or systematic difference. To investigate potential systematic difference between each pair of visits, we performed paired *t*-tests. Furthermore, since magnetic field inhomogeneity between different scanners could lead to the difference of relative BOLD signal intensity (i.e., voxel-level intensity relative to the mean intensity of the whole brain), so we also aimed to investigate to what extent the inter-scanner reliability was influenced by the difference of relative BOLD signal intensity between scanners.

According to the algorithms deriving the three metrics, the relative BOLD signal intensity will affect the three metrics differently. ReHo value and DC value are standardized at voxel-level, i.e., voxel-level ReHo value is from 0 to 1 and DC value of each voxel is -1 ∼ +1. Therefore, ReHo and DC value may not be substantially dependent on the BOLD signal intensity. But, as shown in our previous study (Jia et al., 2017), voxel-level ALFF absolute value is highly dependent on the BOLD signal intensity. Existing solutions include dividing ALFF of each voxel by the global mean ALFF of the whole brain, namely mALFF in the REST software (Song et al., 2011), Z-standardization (minus mean and then divided by the standard deviation of the whole brain) (Yan et al., 2013), and so on. Magnetic field inhomogeneity will affect the mALFF value in the corresponding areas. Therefore, in our previous study, we proposed PerAF, i.e., percent amplitude of fluctuation as a contrast to mean BOLD signal of a single time series, as standardization procedure within a time series (Jia et al., 2017). PerAF could be further standardized by global mean PerAF, i.e., mPerAF (Jia et al., 2017). In the current study, we hypothesized that mPerAF would increase the inter-scanner reliability.

## Materials and Methods

### Participants

Twenty-one healthy participants (21.8 ± 1.8 years old, 11 females) with no history of neurological or psychiatric disorders were recruited. The present study was approved by the Ethics Committee of the Center for Cognition and Brain Disorders (CCBD) at Hangzhou Normal University (HZNU). Written informed consent was obtained from each subject prior to participation.

### Data Acquisition

All subjects were scanned 3 times, with the first two visits (V1, V2, approximately 2 weeks apart) on one GE 3T scanner (MR-750, GE Medical Systems, Milwaukee, WI), located at the CCBD of HZNU. The third visit (V3, about 8 months after V2) was on a Siemens 3T scanner (Prisma, Siemens Healthineers Erlangen, Germany), located at the center for Brain Imaging Science and Technology of Zhejiang University (ZJU). All the raw data will be publicly accessed at https://www.nitrc.org/.

For scans on GE scanner, a gradient echo echo-planar imaging (EPI) pulse sequence was used for BOLD images with following parameters: repetition time (TR) = 2000 ms; echo time (TE) = 30 ms; flip angle (FA) = 90°; 43 slices with interleaved acquisition; matrix = 64 × 64; field of view (FOV) = 220 mm; acquisition voxel size = 3.44 mm × 3.44 mm × 3.20 mm. Moreover, a high resolution T1 anatomical scan was scanned for the spatial normalization (176 sagittal slices, thickness = 1 mm, TR = 8.1 ms, TE = 3.1 ms, FA = 8°, FOV = 250 mm).

For scans on Siemens scanner, the BOLD EPI parameters including TR, TE, FA, slice number, acquisition matrix, and FOV were the same as those obtained from the GE. A high resolution T1 anatomical image was also scanned (176 sagittal slices, thickness = 1 mm, TR = 1800ms, TE = 2.28 ms, FA = 8°, FOV = 250 mm).

For each visit, all the participants underwent two 8-min RS-fMRI sessions, during which they were asked to relax with either EO or EC, not to think of anything in particular, and not to fall asleep. The order of the two sessions was counter-balanced across subjects. To minimize head movement, straps and foam pads were used to fix the head comfortably during scanning.

### Data Preprocessing

Analysis of the RS-fMRI data was performed using DPABI 4.3 toolbox (DPABI_V2.3^1^) (Yan et al., 2016), and Resting-State fMRI Data Analysis Toolkit (RESTplus1.1^2^). The preprocessing included the following procedures: (1) removal of the first 10 volumes; (2) slice timing correction; (3) head motion correction; (4) coregistration of T1 image to the averaged EPI image; (5) spatial normalization to standard Montreal Neurological Institute (MNI) space using “Dartel +segment”; (6) regression of head motion effects with the Friston-24 parameter model. All the subject’s head motion were lower than our criteria of 2 mm and 2°. Additionally, regression of head motion, white matter (WM) and cerebrospinal fluid (CSF) was also performed, and the results were presented in the **Supplementary Material**; (7) removal of linear trends.

### mALFF Calculation

Before ALFF calculation, spatial smoothing (Gaussian kernel of full-width half maximum, FWHM = 6 mm) was performed. Then, with the Fast Fourier Transform (FFT), the time courses of RS-fMRI signal were converted to frequency domain. The averaged square root across a frequency band of 0.01 – 0.08 Hz was calculated as ALFF (Zang et al., 2007). For standardization purpose, ALFF of each voxel was divided by the global mean ALFF, and a mALFF map was obtained.

### mPerAF Calculation

PerAF refers to the percentage of BOLD fluctuation relative to the mean BOLD signal intensity (Jia et al., 2017) of a given time series. After spatial smoothing (Gaussian kernel of full-width half maximum, FWHM = 6 mm) and a band-pass filtering (0.01 – 0.08 Hz), PerAF was calculated. We calculated PerAF as follows (Jia et al., 2017):

(1) $$\begin{array}{c} \mathrm{PerAF}=\frac{1}{n}\overset{n}{\underset{i=1}{\Sigma }}\left|\frac{X_{i}-\mu }{\mu }\right|\times 100 \end{array}$$

(2) $$\begin{array}{c} \mu =\frac{1}{n}\overset{n}{\underset{i=1}{\Sigma }}X_{i} \end{array}$$

where, *X_i_* is the BOLD signal intensity of the *ith* time points, *n* is the total number of time points of a given time series, and μ is the mean intensity of that time series.

Finally, PerAF of each voxel was divided by the global mean PerAF with the Resting-State fMRI Data Analysis Toolkit (RESTplus1.1, see text footnote 2). Hence, a mPerAF map was obtained.

### mReHo Calculation

Before ReHo calculation, band-pass filtering (0.01 – 0.08 Hz) was performed. ReHo was calculated by using Kendall coefficient of concordance (KCC) as the following formula (Zang et al., 2004):

(3) $$\begin{array}{c} w=\frac{\sum_{i=1}^{n}{\left(R_{i}\right)}^{2}-n{\left({\bar{R}}_{i}\right)}^{2}}{\frac{1}{12}K^{2}\left(n^{3}-n\right)} \end{array}$$

where w is the KCC (ranged from 0–1) of given 27 nearest neighboring voxels was assigned to the center voxel. *K* is the number neighboring voxels (here, *K* = 27, including the center voxel), ${\bar{R}}_{i}$ is the mean rank across nearest neighbors (27 voxels) at the *ith* time point, *n* is the total number of time points of the time series. For standardization purpose, each voxel’s ReHo value was divided by the global mean ReHo, and hence a mReHo map was obtained. Spatial smoothing (FWHM = 6 mm) was performed after the ReHo calculation.

### mDC Calculation

Before DC calculation, band-pass filtering (0.01 – 0.08 Hz) was performed. DC represents the functional strength of a given voxel with all voxels in the brain. We calculated the Pearson correlation of the time series of a given voxel with that of each voxel in the whole brain. It should be noted that a previous study has shown that binary DC and weighted DC were highly similar (Liao et al., 2013). Then binary Pearson correlation coefficient was used with a threshold of 0.25. Then the summed value was assigned to that given voxel. Voxel-wise whole-brain DC map was obtained. For standardization purpose, each voxel’s DC was divided by the global mean DC, then a mDC map was obtained (Zuo et al., 2012). Then, spatial smoothing was performed (FWHM = 6 mm).

### Relative BOLD Signal Intensity

Relative BOLD signal intensity in the current study was the voxel-level signal intensity relative to the mean signal intensity of the whole brain. After normalization, the BOLD signal intensity of each voxel in the mean EPI image (over 230 time points) was divided by the global mean BOLD signal intensity of that image. Hence, a relative BOLD signal intensity image was obtained.

### Intra-Class Correlation Coefficient (ICCs)

The intra-scanner (i.e., V1 vs. V2) and inter-scanner (i.e., V1 vs. V3 and V2 vs. V3) reliability of the metrics including of mALFF, mReHo, mDC, and mPerAF were estimated using ICC for EO and EC, respectively, in a way of VWWB analysis according to the following equation (Shrout and Fleiss, 1979):

(4) $$\begin{array}{c} \mathrm{ICC}=\frac{MSb-MSw}{MSb+\left(K-1\right)MSw} \end{array}$$

where, *MSb* represents between-subject effect, *MSw* represents within-subject effect, and *K* is the number of sessions.

To view the regions with moderate or higher reliability, a threshold of ICC > = 0.4 was used to generate ICC maps. Further, a histogram of all voxels of each ICC map was plotted to visually compare the intra- or inter-scanner reliability among metrics and between EO and EC conditions. In addition, the ICC was again calculated while regressing out head motion, WM and CSF. The results with regression were very similar with the results of without regression (see the **Supplementary Material**).

### Paired *t*-Test Between Each Pair of Visits

To investigate the difference of each pair of visits, we performed paired *t*-test on mALFF, mReHo, mDC, mPerAF, and relative BOLD signal intensity maps (i.e., voxel-level intensity relative to the mean intensity of the whole brain). In addition, to account for confounding effects, head motion, WM and CSF were regressed out in the preprocessing stage. Further, sex, age, and interval days between each pair of visits were taken as covariates when performing paired *t*-tests. The results with regression were very similar with the results of without regression (see the **Supplementary Material**). It should be noted that the purpose of the paired *t*-test was to find potential differences. Therefore, a voxel level *p* < 0.05 was used without multiple comparison correction.

## Results

### Intra- and Inter-Scanner Reliability

**FIGURE 1:**
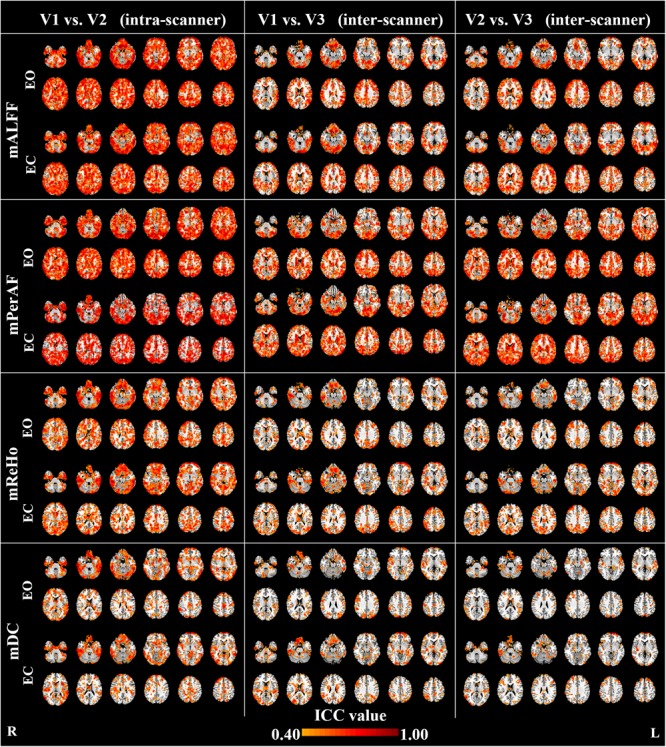
The intra- and inter-scanner reliability of mALFF, mPerAF, mReHo and mDC of eyes open (EO) and eyes closed (EC). The Z coordinates were from –36 to +52 with a step of 8 mm. ICC: intra-class correlation. V: visit.

**FIGURE 2:**
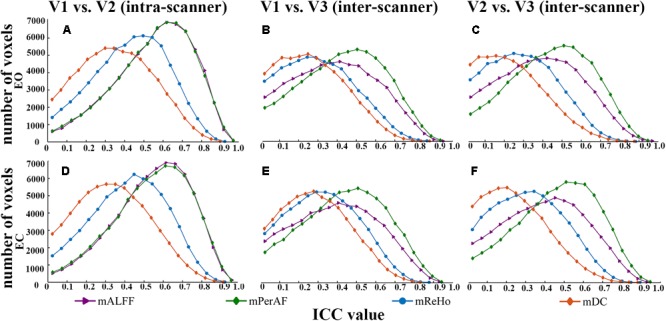
The comparison of reliability histogram among metrics of EO and EC. Intra-scanner reliability: **(A,D)**; Inter-scanner reliability: **(B,C,E,F)**. ICC: intra-class correlation. V: visit.

**FIGURE 3:**
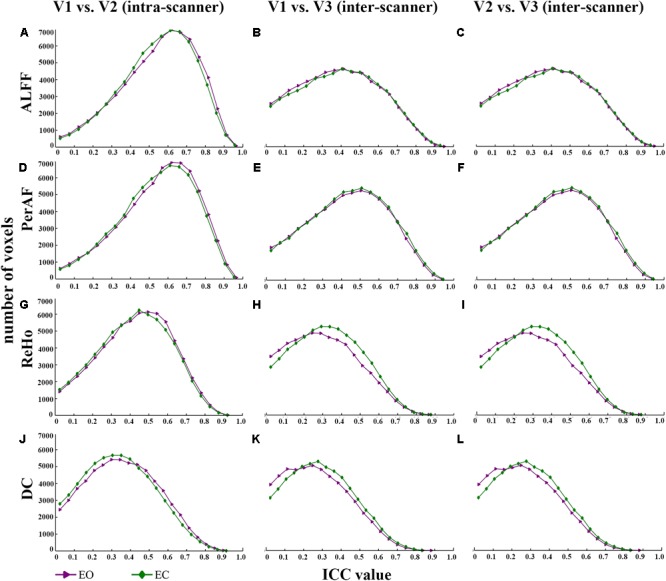
The comparison of reliability histogram between EO and EC. Intra-scanner reliability: **(A,D,G,J)**; Inter-scanner reliability: **(B,C,E,F,H,I,K,L)**. ICC: intra-class correlation. V: visit.

Maps of intra- and inter-scanner reliability of the VWWB metrics were shown in **Figure 1**. The reliability histograms were shown in **Figures 2, 3**. The number of voxels with ICC > = 0.4 for each metric in each condition was shown in **Table 1**. Overall, the intra-scanner reliability was higher than the inter-scanner reliability of all the 4 VWWB metrics under both EO and EC conditions. Moreover, gray matter showed higher both intra- and inter-scanner reliability than the WM for all the 4 VWWB metrics (**Figure 1**).

**Table 1:** The number of voxels with ICC > = 0.4 (with head motion regression).

	The number of voxels with ICC > = 0.4 (with head motion regression)			
		V1 vs. V2 (intra-scanner)	V1 vs. V3 (inter-scanner)	V2 vs. V3 (inter-scanner)
mALFF	EO	53992	28553	29057
	EC	53946	29917	31105
mPerAF	EO	53896	37072	39002
	EC	42670	38421	42158
mReHo	EO	39018	18157	17058
	EC	37366	21867	20422
mDC	EO	26763	13311	9608
	EC	24030	16410	10541

*ICC: intra-class correlation. EO: eyes open. EC: eyes closed. V: visit.*

Summarized comparisons of reliability were as follows:

(I) Intra-scanner reliability > inter-scanner reliability (for all metrics) (**Figure 2** and **Table 1**);
(I) Intra-scanner reliability: mPerAF ≈ mALFF > mReHo > mDC (**Figure 2** and **Table 1**);
(III) Inter-scanner reliability: mPerAF > mALFF > mReHo > mDC (**Figure 2** and **Table 1**);
(IV) EO ≈ EC (all metrics) (**Figure 3** and **Table 1**).

### Intra- and Inter-Scanner Difference

**FIGURE 4:**
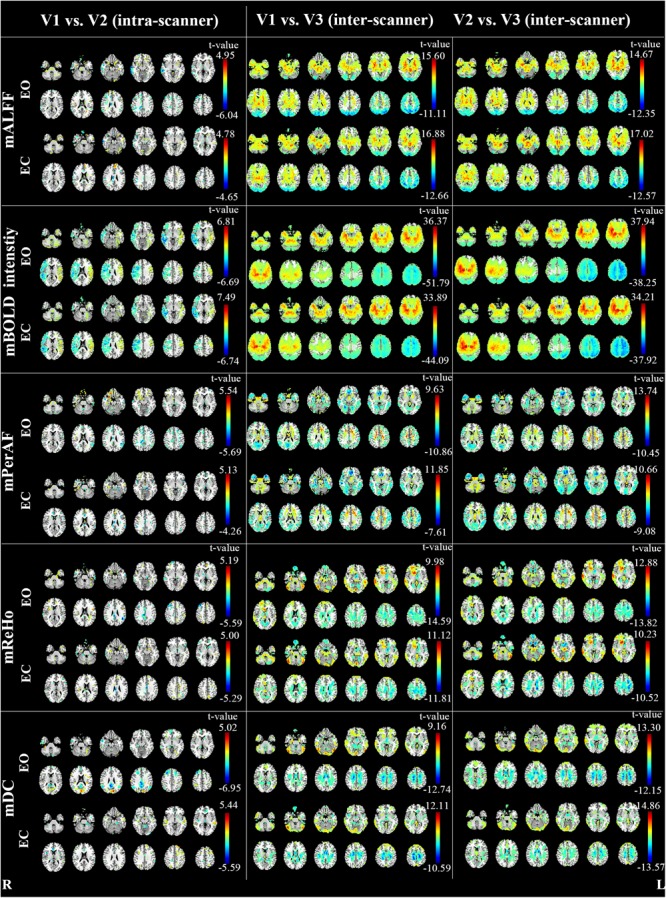
The intra- and inter-scanner difference of mALFF, mPerAF, mReHo and mDC of EO and EC (*p* < 0.05, uncorrected). The Z coordinates were from –36 to +52 with a step of 8 mm. V: visit.

The inter-scanner difference appears larger than the intra-scanner difference for all the four metrics (**Figure 4**).

As for the intra-scanner difference of mALFF under EO, a few clusters in the right hemisphere showed significant lower mALFF for V1 than V2 (**Figure 4**). As for the inter-scanner difference, V1 and V2 showed significantly higher mALFF than V3 in large part of the inferior and anterior brain regions, while showed significantly lower mALFF than V3 in large part of superior and posterior brain regions. By visual inspection, the inter-scanner difference patterns were similar for V1-V3 and V2-V3 under both EO and EC (**Figure 4**). The relative BOLD signal intensity (i.e., voxel-level intensity relative to the mean intensity of the whole brain) in some brain areas also showed significant intra-scanner difference (**Figure 4**). Specifically, the right hemisphere of V1 showed lower relative BOLD signal intensity than V2, while the left hemisphere showed higher relative BOLD signal intensity for V1 than V2, under both EO and EC (**Figure 4**).

Notably, as shown in **Figure 4**, the inter-scanner differences of the relative BOLD signal intensity were very similar with that of inter-scanner mALFF differences (V1 vs. V3 and V2 vs. V3), but not with that of mReHo or mDC.

## Discussion

### Reliability of Metrics

The results of moderate to high intra-scanner reliability (i.e., test-retest reliability) of mALFF, mPerAF, mReHo, and mDC were consistent with previous studies (Zuo et al., 2010c, 2012, 2013; Li et al., 2012; Somandepalli et al., 2015; Jia et al., 2017). Zuo and Xing (2014) systematically investigated the test-retest reliability (i.e., intra-scanner reliability) of ALFF, ReHo and DC. They found that DC displayed the worst reliability, being consistent with our findings. As for comparison between ALFF and ReHo, Zuo and Xing found slightly better test-retest reliability of ReHo than ALFF, while Somandepalli and colleagues found that the reliability of ALFF was slightly greater than ReHo (Somandepalli et al., 2015). We also found slightly better reliability of ALFF than ReHo. In summary, ALFF and ReHo shows similar reliability, while both ALFF and ReHo shows much higher reliability than DC.

Our previous study had suggested that the number of voxels with ICC > 0.5 of mPerAF were slightly larger than that of mALFF (number of voxels for short-term reliability: 46336 vs. 44089 voxels; long-term reliability: 31248 vs. 30866 voxels) (Jia et al., 2017). In the current study, we found that the mALFF was similar to mPerAF in intra-scanner reliability, but mPerAF was better than mALFF in inter-scanner reliability (**Figure 2** and **Table 1**). For standardization purpose, ALFF was usually divided by the mean ALFF of the entire brain, i.e., mALFF (Zang et al., 2007). Such standardization procedure seemed work well for different scanning sessions in the same scanner. However, as shown in **Figure 4**, the relative BOLD signal, i.e., the mean BOLD signal divided by that of the entire brain, was significantly different between the Siemens and GE scanners. The spatial pattern of mALFF difference between the two scanners was very similar with the spatial pattern of relative BOLD signal difference (**Figure 4**). As compared with mALFF, mPerAF has two stages of standardization (Jia et al., 2017). The first stage is percent amplitude of fluctuation at single voxel or signal time series level. The second stage is similar as that of mALFF, i.e., divided by the mean PerAF of the entire brain. While the intra-scanner reliability was almost the same for mALFF and mPerAF, the inter-scanner reliability of mPerAF was slightly higher than mALFF. By simulation, it was shown that the ALFF was affected by the mean value of BOLD signal intensity, but PerAF was not (Jia et al., 2017). The relative BOLD signal intensity of the two visits on the same scanner was very similar, however, was very different for the two visits on two different scanners. The better inter-scanner reliability of mPerAF over mALFF suggests that mPerAF could calibrate the variation brought by the difference of relative BOLD signal intensity of different scanners.

### Reliability of Eyes Open (EO) vs. Eyes Closed (EC) Conditions

In RS-fMRI studies, EO, EC, and EO with fixation (EO-F) are three widely used awake conditions. Although Fox and colleagues reported that the FC pattern of the default mode network (DMN) was very similar across the three conditions (Fox et al., 2005), Yan et al. (2009) found that the local activity (including ALFF) and the FC were significantly different among the three conditions in the DMN as well as in the sensorimotor cortex and visual cortex. The difference between EO and EO-F was not as big as the difference between EO and EC with or without fixation (Yan et al., 2009). Therefore, similar to a few previous studies (Liu et al., 2013; Yuan et al., 2014; Zou et al., 2015), the current study included only EO and EC conditions but did not include EO-F condition. However, Patriat and colleagues investigated the test-retest reliability of the three conditions and concluded that, overall, EO-F had the highest test-retest reliability of FC (Patriat et al., 2013). It should be noted that Patriat and colleagues only investigated networks with significant connectivity, but not the whole brain. Future study should pay attention on the test-retest reliability of the VWWB metrics, i.e., mALFF, mPerAF, mReHo, and mDC of RS-fMRI with all three conditions (EO, EC, and EO-F). But it should keep in mind that EO-F is, at least as compared with EO and EC, a certain task condition. It requires the participant to cooperate as much as possible during scanning. While such cooperation might be easily achievable for young adult volunteers, it might be a cognitive burden for other participants, especially patients. Therefore, for a patient study, it should be cautious to use only EO-F as the RS-fMRI scanning condition.

As for the comparison of test-retest reliability between EO and EC, Zou and colleagues reported that EO showed slightly higher test-retest reliability than EC for mALFF (Zou et al., 2015). In the current study, we found that EO and EC showed very similar reliability, both for intra-scanner (i.e., test-retest) and inter-scanner comparisons.

### ICC vs. Paired *t*-Test

Most reliability studies of RS-fMRI have utilized ICC. But lower ICC could be due to both random variance and systematic variance. Therefore, we performed paired t-test between each pair of two visits. As expected, we found very significant between-scanner differences for all metrics. The brain regions showing significant between-scanner differences were largely overlapped with the brain regions showing lower inter-scanner reliability, especially in the WM. Such systematic difference was the most prominent for mALFF. As discussed in the section of “4.1. Reliability of metrics”, it might be due to the computational limitation of mALFF. To some extent, mPerAF reduced such systematic difference. We therefore recommend mPerAF over mALFF in future studies.

We found small systematic difference by intra-scanner paired *t*-test for mALFF, mPeAF, mReHo, and mDC. The areas showing lower ICC did not show significant difference by the paired *t*-test. It means the lower ICC in these areas might be due to random variance between the two visits on the same scanner.

### Limitations

There were a few limitations. First, because we intended to investigate both intra- and inter-scanner reliability, the order of the two visits of inter-scanner reliability was unable to be randomized. If a study aims to investigate only the inter-scanner reliability, the order of the two visits should be counter-balanced. Second, the current study only investigated VWWB metrics of RS-fMRI. Future studies should also investigate the inter-scanner reliability of other metrics. Third, in order to keep consistent among metrics in our study, we used the same standardization procedure of “dividing global mean value” for all metrics. However, it has been reported that the standardization procedure could affect the test-retest reliability of ALFF, ReHo, and DC differently (Yan et al., 2013). Therefore, the standardization procedure should be further investigated.

## Conclusion

The inter-scanner reliability was much lower than intra-scanner reliability. For all the 4 metrics of RS-fMRI, mDC showed the lowest intra- and inter-scanner reliability. mPerAF showed similar intra-scanner reliability as mALFF, but showed increased inter-scanner reliability over mALFF. We thus recommend using mPerAF for future studies. Measurements under eyes open and eyes closed conditions showed very similar reliability. Paired *t*-test may provide additional information for studies on either intra-scanner or inter-scanner reliability.

## Author Contributions

NZ, JW, and Y-FZ analyzed the data and wrote the paper. L-XY collected and processed the data. X-ZJ, X-FZ, and X-PD processed the data. JZ and H-JH collected the data. All authors designed the experiments.

## Conflict of Interest Statement

The authors declare that the research was conducted in the absence of any commercial or financial relationships that could be construed as a potential conflict of interest.

## Footnotes

1 http://rfmri.org/dpabi

2 http://www.restfmri.net
