# Supplementary material for: Intra- and Inter-Scanner Reliability of Voxel-Wise Whole-Brain Analytic Metrics for Resting State fMRI
Source: Front Neuroinform. 2018 Aug 21;12:54. doi: 10.3389/fninf.2018.00054 (PMC6110941; doi:10.3389/fninf.2018.00054)
Supplement: Supplementary file 1 [file Data_Sheet_1.DOCX]

Supplementary Material

## Intra- and inter-scanner reliability of voxel-wise whole-brain analytic metrics for resting state fMRI

**Na Zhao^1,2^, Li-Xia Yuan^3^, Xi-Ze Jia^1,2^, Xu-Feng Zhou^1,2^, Xin-Ping Deng^1,2^, Hong-Jian He^3^, Jianhui Zhong^3^, Jue Wang^1,2*^, Yu-Feng Zang^1,2*^**

^1^Center for Cognition and Brain Disorders, Institutes of Psychological Sciences, Hangzhou Normal University, Hangzhou, China

^2^Zhejiang Key Laboratory for Research in Assessment of Cognitive Impairments, Hangzhou, China

^3^Center for Brain Imaging Science and Technology, Key Laboratory for Biomedical Engineering of Ministry of Education, College of Biomedical Engineering and Instrumental Science, Zhejiang University, Hangzhou, China

**Correspondence:**

Jue Wang

juefirst@163.com

Yu-Feng Zang

zangyf@hznu.edu.cn

1. **Supplementary Figures and Tables**

The number of voxels with ICC>=0.4 (with head motion, WM and CSF regression).

|  | The number of voxels with ICC>=0.4（with head motion, WM, CSF regression） | | | |
| --- | --- | --- | --- | --- |
|  |  | V1 vs. V2 (intra-scanner) | V1 vs. V3 (inter-scanner) | V2 vs. V3 (inter-scanner) |
| mALFF | EO | 52388 | 26858 | 27837 |
|  | EC | 51722 | 27050 | 29189 |
| mPerAF | EO | 52489 | 34447 | 37017 |
|  | EC | 52327 | 34712 | 39721 |
| mReHo | EO | 36728 | 17651 | 17756 |
|  | EC | 34707 | 20280 | 15591 |
| mDC | EO | 25022 | 14245 | 11546 |
|  | EC | 25674 | 15164 | 12825 |

ICC: intra-class correlation. eyes open: EO, eyes closed: EC. V: visit. WM: white matter. CSF: cerebrospinal fluid

- 1. **Supplementary Figures**

**
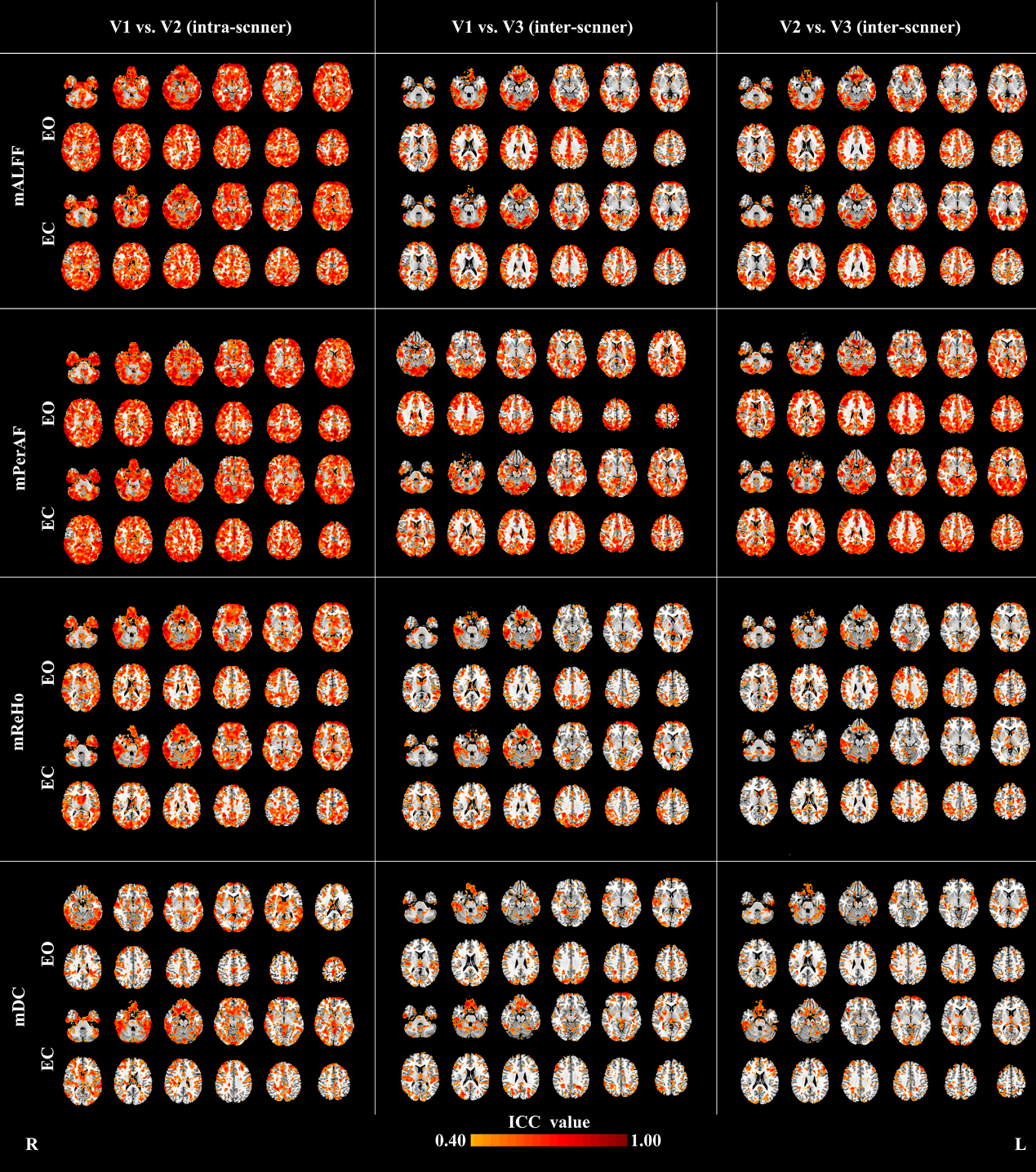
 Supplementary Figure 1.** The intra- and inter-scanner reliability of mALFF, mPerAF, mReHo and mDC of eyes open (EO) and eyes closed (EC) with head motion, white matter (WM) and cerebrospinal fluid (CSF) as covariates. The Z coordinates were from -36 to +52 with a step of 8 mm. ICC: intra-class correlation. V: visit.


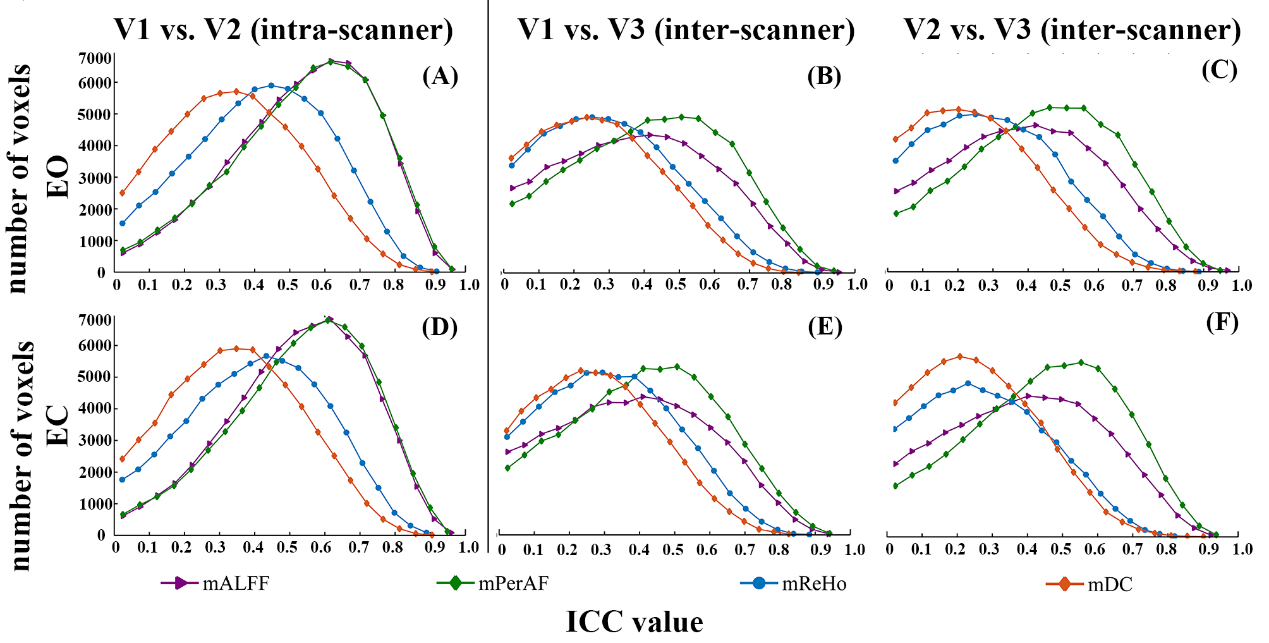


**Supplementary Figure 2.** The comparison of reliability histogram among metrics of eyes open (EO) and eyes closed (EC) with head motion, white matter (WM) and cerebrospinal fluid (CSF) as covariates. Intra-scanner reliability: (A), (D); Inter-scanner reliability: (B), (C), (E), (F). ICC: intra-class correlation. V: visit.


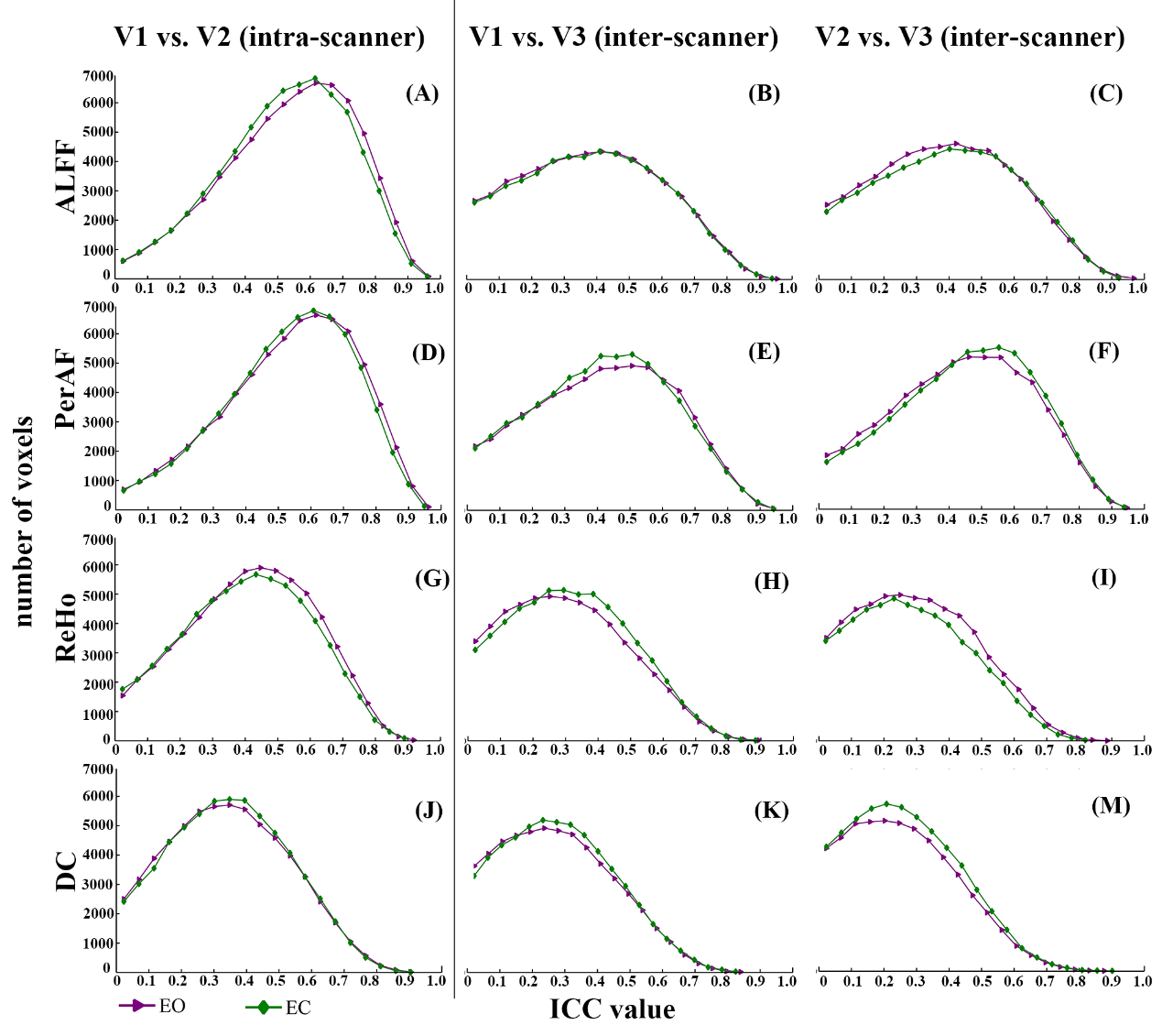


**Supplementary Figure 3.** The comparison of reliability histogram between eyes open (EO) and eyes closed (EC) with head motion, white matter (WM) and cerebrospinal fluid (CSF) as covariates. Intra-scanner reliability: (A), (D), (G), (J); Inter-scanner reliability: (B), (C), (E), (F), (H), (I), (K), (M). ICC: intra-class correlation. V: visit.


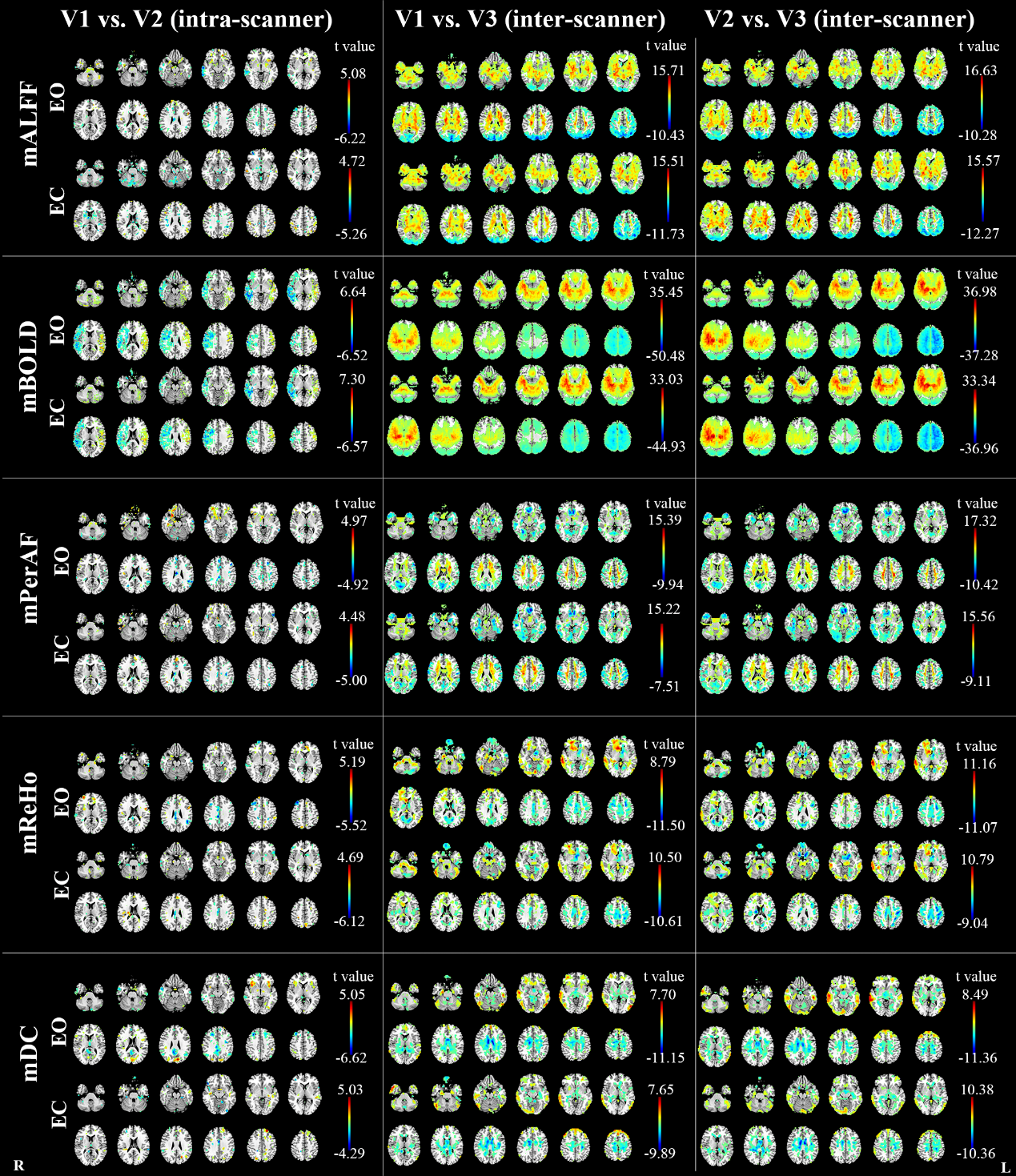


**Supplementary Figure 4.** The intra- and inter-scanner difference of mALFF, mPerAF, mReHo and mDC of eyes open (EO) and eyes closed (EC) (*p* < 0.05, uncorrected) with head motion, white matter (WM) and cerebrospinal fluid (CSF) as covariates. The Z coordinates were from -36 to +52 with a step of 8 mm. V: visit.
